# Characterizing chromatin interactions of regulatory elements and nucleosome positions, using Hi-C, Micro-C, and promoter capture Micro-C

**DOI:** 10.1186/s13072-022-00473-4

**Published:** 2022-12-21

**Authors:** Beoung Hun Lee, Zexun Wu, Suhn K. Rhie

**Affiliations:** Department of Biochemistry and Molecular Medicine and the Norris Comprehensive Cancer Center, Keck School of Medicine, University of Southern California, Los Angeles, CA 90089 USA

**Keywords:** Chromatin loops, Regulatory elements, Nucleosomes, Epigenomics, Human cancer cells

## Abstract

**Background:**

Regulatory elements such as promoters, enhancers, and insulators interact each other to mediate molecular processes. To capture chromatin interactions of regulatory elements, 3C-derived methods such as Hi-C and Micro-C are developed. Here, we generated and analyzed Hi-C, Micro-C, and promoter capture Micro-C datasets with different sequencing depths to study chromatin interactions of regulatory elements and nucleosome positions in human prostate cancer cells.

**Results:**

Compared to Hi-C, Micro-C identifies more high-resolution loops, including ones around structural variants. By evaluating the effect of sequencing depth, we revealed that more than 2 billion reads of Micro-C are needed to detect chromatin interactions at 1 kb resolution. Moreover, we found that deep-sequencing identifies additional long-range loops that are longer than 1 Mb in distance. Furthermore, we found that more than 50% of the loops are involved in insulators while less than 10% of the loops are promoter–enhancer loops. To comprehensively capture chromatin interactions that promoters are involved in, we performed promoter capture Micro-C. Promoter capture Micro-C identifies loops near promoters with a lower amount of sequencing reads. Sequencing of 160 million reads of promoter capture Micro-C resulted in reaching a plateau of identifying loops. However, there was still a subset of promoters that are not involved in loops even after deep-sequencing. By integrating Micro-C with NOMe-seq and ChIP-seq, we found that active promoters involved in loops have a more accessible region with lower levels of DNA methylation and more highly phased nucleosomes, compared to active promoters that are not involved in loops.

**Conclusion:**

We determined the required sequencing depth for Micro-C and promoter capture Micro-C to generate high-resolution chromatin interaction maps and loops. We also investigated the effect of sequencing coverage of Hi-C, Micro-C, and promoter capture Micro-C on detecting chromatin loops. Our analyses suggest the presence of distinct regulatory element groups, which are differently involved in nucleosome positions and chromatin interactions. This study does not only provide valuable insights on understanding chromatin interactions of regulatory elements, but also present guidelines for designing research projects on chromatin interactions among regulatory elements.

**Supplementary Information:**

The online version contains supplementary material available at 10.1186/s13072-022-00473-4.

## Background

Chromatin interactions have been studied using chromatin conformation capture (3C) assay and its derivatives such as 4C, 5C, ChIA-PET, HiChIP, and Hi-C [1–6]. Specifically, Hi-C has been one of the most popular methods to study genome-wide chromatin interactions [7]. Hi-C assay has been useful in studying chromatin compartmentalization, topologically associating domains (TADs), and chromatin interactions [6, 8]. However, there are challenges on identifying high-resolution chromatin interactions from Hi-C. Because Hi-C uses restriction enzymes that give a bias on fragmentation of chromatin, the coverage of Hi-C is not comprehensive and uniform. Therefore, the average chromatin fragment size of Hi-C is 4 kb, which is bigger than most of regulatory elements [9].

Regulatory elements are reported to make loops to mediate molecular processes. Regulatory elements are identified using chromatin immunoprecipitation followed by sequencing (ChIP-seq) with antibodies targeting specific histone modifications or proteins (e.g., H3K4me3 for promoters, H3K27ac for enhancers, CTCF for insulators, H3K27me3 for repressed regions, and H3K9me3 for heterochromatin regions) [10]. The size of identified regulatory elements vary, but the average size of active regulatory elements such as enhancers and insulators is less than 2 kb [11–13]. Chromatin accessibility assays such as DNase-seq, ATAC-seq (Assay of Transposase Accessible Chromatin sequencing), and NOMe-seq (Nucleosome Occupancy and Methylome sequencing) identify nucleosome-depleted regions (NDRs) where transcription factors bind [10]. The average size of the identified NDRs is smaller than 1 kb [14, 15]. Due to the size, it is difficult to detect comprehensive chromatin loops of regulatory elements and NDRs using Hi-C.

To overcome this limitation, recent studies have developed Micro-C, a novel method to study chromatin interactions at single nucleosome-resolution using Micrococcal nuclease (MNase), which cleaves DNA around nucleosomes uniformly, yielding smaller fragment sizes when compared to restriction enzyme digestion [9, 16, 17]. These studies have shown that Micro-C improved identifying chromatin interactions at higher resolution compared to Hi-C. Moreover, it is suggested to perform targeted sequencing (e.g., capture Micro-C) to reduce the sequencing cost to map chromatin interactions for the regions of interest. However, there are many questions needed to be addressed when designing experiments to study chromatin loops. For example, it is not yet clear how many sequencing reads and libraries of Micro-C and capture Micro-C are required to identify high-resolution chromatin interactions in human cells. It is not yet characterized how sequencing depth of Micro-C affects the identification of chromatin loops. Furthermore, it is not yet comprehensively determined which chromatin loops of regulatory elements and NDRs can be captured using Micro-C.

To address these, here we performed Hi-C, Micro-C, and promoter capture Micro-C experiments in human prostate cancer cells. In detail, we compared Hi-C, Micro-C, and promoter capture Micro-C datasets and investigated the effect of sequencing depth on identifying global chromatin interactions. Moreover, we integrated chromatin interaction datasets with ChIP-seq datasets to investigate chromatin loops that involve regulatory elements such as promoter–enhancer loops. Furthermore, by analyzing them with NOMe-seq that independently maps nucleosome occupancy and DNA methylation levels at single molecule resolution, we assessed the involvement of nucleosome positions in chromatin interactions.

## Results

### Micro-C captures more chromatin interactions than Hi-C

**Fig. 1:**
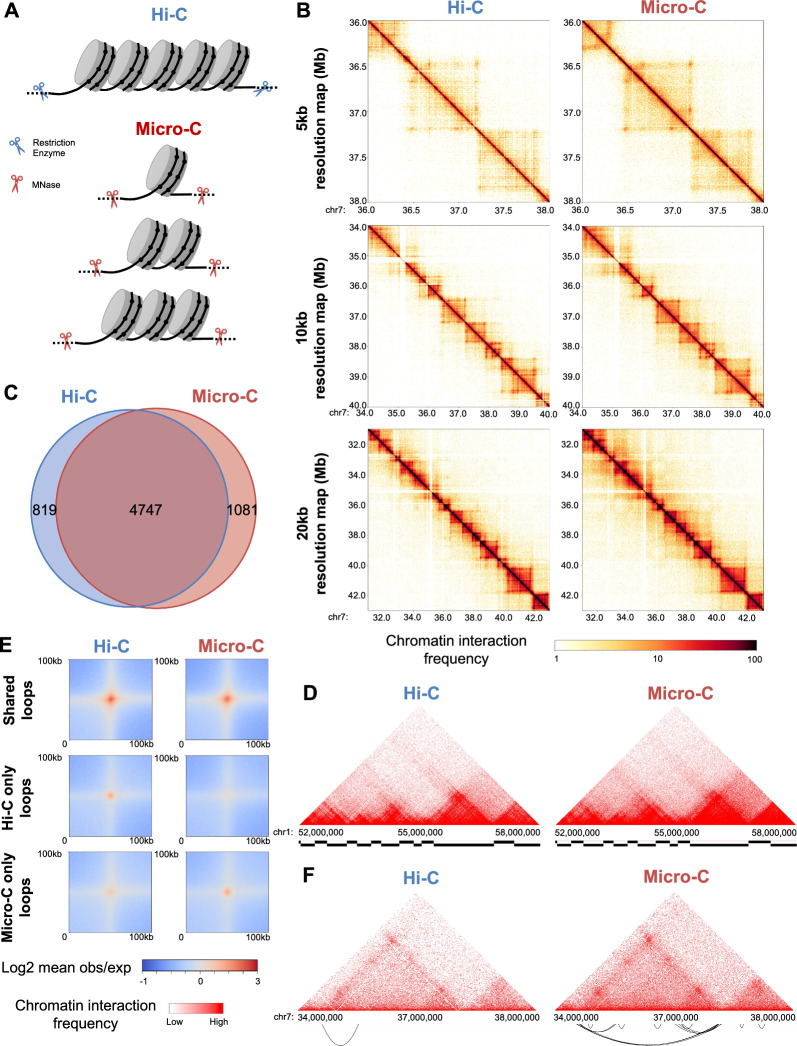
Comparison of Hi-C and Micro-C data. **A** Experimental methods of Hi-C and Micro-C. Unlike Hi-C that uses restriction enzyme, Micro-C uses MNase, allowing to fragment chromatin to mono, di-, and tri-nucleosomes. **B** Chromatin interaction heatmaps of Hi-C and Micro-C data near chr7p14 region. **C** Venn diagram of TADs identified from Hi-C and Micro-C. **D** Triangular heatmaps of Hi-C and Micro-C near chr1p32 region. TADs identified from each data are shown at the bottom. **E** Average chromatin interaction signals at shared loops (loops found in Hi-C and Micro-C) and unique loops are shown. **F** Triangular heatmaps of Hi-C and Micro-C near chr7p14 region and loops identified from each data are shown at the bottom

To identify comprehensive chromatin interactions in human prostate cancer cells, we performed Hi-C and Micro-C in C42B prostate cancer cells. While Hi-C uses restriction enzymes that cleave specific sequences, Micro-C uses MNase to digest cross-linked DNA in regions that are not stably bound by proteins across the genome. Therefore, Hi-C can result in multi-nucleosome-sized fragments while Micro-C resulted in mono, di, or tri-nucleosome sized fragments (Fig. 1A). After generating multiple replicates of Hi-C and Micro-C datasets, we sequenced Hi-C and Micro-C data at total 1 billion read pairs per data (Hi-C 1 billion data: total 1,094,888,777 raw read pairs, Micro-C 1 billion data: total 1,050,616,368 raw read pairs) (Additional file 1: Table S1A). Hi-C and Micro-C reads are mapped to the genome using BWA MEM [18]; 79% of Micro-C reads were mapped to the genome, and 61% of Hi-C reads were mapped to genome (Additional file 1: Table S1). Starting from the total 1 billion read pairs, after removing duplicates (PCR duplicates % for Hi-C 1 billion data: 9.78%, Micro-C 1 billion data: 15.36%) and invalid ligated reads using Pairtools (https://pairtools.readthedocs.io/en/latest), Micro-C had 560 million valid read pairs, and Hi-C had 433 million valid read pairs. Micro-C valid read pairs also included similar percentage of trans read pairs (pairs between different chromosomes) and cis read pairs (pairs between the same chromosome) (Additional file 1: Table S1). To compare the coverage, we generated 1 kb, 2 kb, 4 kb, 5 kb, 10 kb, and 20 kb resolution chromatin interaction heatmaps using Hi-C 1 billion data and Micro-C 1 billion data (Fig. 1B, Additional file 2: Figure S1). Hi-C and Micro-C data showed a comparable number of interactions at lower resolutions like 20 kb. However, when we compared Hi-C 1 billion data and Micro-C 1 billion data at higher resolutions, such as 5 kb and 2 kb resolutions, Micro-C showed better coverage than Hi-C (Fig. 1B, Additional file 2: Figure S1). However, at 1 kb resolution, both Hi-C 1 billion data and Micro-C 1 billion data displayed less interactions, indicating that 1 billion read pairs were not enough to detect chromatin interactions at 1 kb resolution.

Next, we identified TADs using TopDom program (Shin et al., 2016) from Hi-C 1 billion data and Micro-C 1 billion data at 50 kb resolution. We identified similar numbers of TADs from Hi-C and Micro-C data (Hi-C: 5,566 vs Micro-C: 5,828) (Additional file 3: Table S2). Hi-C and Micro-C displayed similar patterns of TADs that are about 450 kb sized, and identified TADs are mostly shared between datasets (Fig. 1C, D). When we compared the number of identified chromatin loops using Mustache program [19], Hi-C 1 billion data and Micro-C 1 billion data identified a similar number of loops at 10 kb resolution (Hi-C: 25,377 vs Micro-C: 25,502), 25 kb resolution (Hi-C: 13,216 vs Micro-C: 12,890), and 50 kb resolution (Hi-C: 6,141 vs Micro-C: 6,407) (Additional file 4: Table S3). However, at higher resolutions such as 5 kb resolution, Hi-C data identified 22,945 loops while Micro-C data identified 28,390 loops; additional 5,000 loops were identified in Micro-C data (Additional file 4: Table S3). When we compared loops, 12,531 loops are commonly found in both Hi-C and Micro-C (shared loops), 10,414 loops are found exclusively in Hi-C (Hi-C only loops) and 15,386 loops are found exclusively in Micro-C (Micro-C only loops) (Fig. 1E). Micro-C identified more loops than Hi-C at 2 kb resolution (Hi-C: 4,429 vs Micro-C: 7,744) and 1 kb resolution (Hi-C: 199 vs Micro-C: 909) (Additional file 4: Table S3). For example, we were able to detect more robust loops from Micro-C 1 billion data than Hi-C 1 billion data at chr7q14 region (Fig. 1F).

### More than 2 billion reads of Micro-C are needed to capture chromatin interactions at 1 kb resolution

**Fig. 2:**
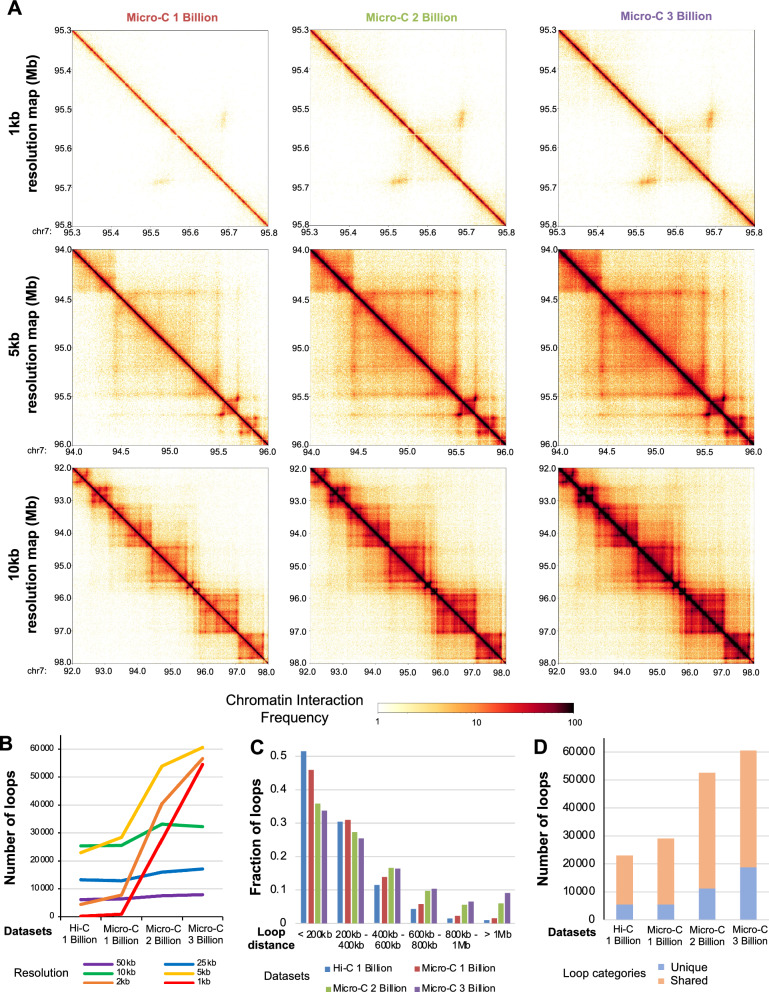
Comparison of Micro-C data in different read depth sequencing. **A** Chromatin interaction heatmaps of Micro-C 1 billion, 2 billion, and 3 billion data near chr7q21 region. Heatmaps are generated at 1 kb resolution (top), 5 kb resolution (middle), and 10 kb resolution (bottom). **B** Numbers of loops identified by Mustache at different resolutions from Hi-C 1 billion, Micro-C 1 billion, Micro-C 2 billion, and Micro-C 3 billion data are shown. **C** Fractions of loops that have different lengths (distances) found from Hi-C 1 billion, Micro-C 1 billion, 2 billion, and 3 billion data are shown. **D** Numbers of loops shared (between any datasets) or unique among Hi-C 1 billion, Micro-C 1 billion, 2 billion, and 3 billion data are shown

While Micro-C 1 billion data analysis identified more chromatin interactions at higher resolution compared to Hi-C 1 billion data, it still identified a small amount of chromatin interactions at 2 kb or higher resolution. Therefore, we generated additional libraries (total 16 libraries) and sequenced Micro-C data to have 2 billion and 3 billion raw read pairs and performed comparison analysis (Additional file 1: Table S1). Similar to Micro-C 1 billion data, both 2 billion and 3 billion data had about 80% of its reads aligned to the genome. After removing PCR duplicates (PCR duplicates % for Micro-C 1 billion data: 15.36%, Micro-C 2 billion data: 16.44%, Micro-C 3 billion data: 18.43%) and invalid read pairs, we used valid read pairs of Micro-C 1 billion data (560 million valid read pairs), 2 billion data (1.33 billion valid read pairs) and 3 billion data (1.89 billion valid read pairs) for downstream analyses (Additional file 1: Table S1). Micro-C 1 billion, 2 billion, and 3 billion data identified over 5,800 TADs which are mostly shared (90%) among datasets (Additional file 3: Table S2). Micro-C 1 billion, 2 billion, and 3 billion data showed comparable heatmaps and chromatin interaction patterns at lower resolutions, but Micro-C 2 billion and 3 billion data showed much stronger interaction signals at 1 kb resolution and identified more chromatin interactions that were not seen in Micro-C 1 billion data (Fig. 2A).

To comprehensively compare chromatin loops identified by Hi-C 1 billion, Micro-C 1 billion, 2 billion, and 3 billion data at different resolutions, we identified chromatin loops at 1 kb, 2 kb, 5 kb, 10 kb, 25 kb, and 50 kb-binned matrices of data using Mustache [19], SIP [20], and HiCCUPS [21] loop calling programs (Fig. 2B, Additional file 2: Figure S2A and S2B and Figure S3). At 50 kb, 25 kb, and 10 kb resolutions, all of the datasets identified a comparable number of chromatin loops from all loop calling programs (Additional file 4: Table S3). However, starting from 5 kb resolution, Micro-C 2 billion data and 3 billion data identified more chromatin loops than Micro-C 1 billion data. For example, Micro-C 2 billion data (2 kb resolution: 27,554, 1 kb resolution: 40,533) and 3 billion data (2 kb resolution: 566,22, 1 kb resolution: 54,506) identified substantially more chromatin loops at 1 kb and 2 kb resolutions, compared to Hi-C 1 billion data (2 kb resolution: 4,429, 1 kb resolution: 199) and Micro-C 1 billion data (2 kb resolution: 7,744, 1 kb resolution: 909) (Fig. 2B, Additional file 4: Table S3). This pattern was also consistent among loop calling programs (Additional file 4: Table S3, Additional file 2: Figure S2A and S2B), indicating that more than 2 billion reads of Micro-C are needed to capture chromatin interactions at 1 kb resolution.

### Deeply sequenced Micro-C data identifies additional long-range loops that are not detected from relatively lowly sequenced data

Because Mustache program identified the greatest number of loops and the identified loops are largely shared with the loops from other loop calling programs, the loops identified from Mustache have been used in further analysis (Additional file 2: Figure S2C). Next, we investigated if there is any difference on the distance of loops identified by datasets by categorizing loops to shorter distanced to longer-range loops (< 200 kb, 200 kb–400 kb, 400 kb–600 kb, 600 kb–800 kb, 800 kb–1 Mb, > 1 Mb). Interestingly, we found that Micro-C 2 billion data and 3 billion data called more long-range loops than Hi-C 1 billion data and Micro-C 1 billion data (Fig. 2C, Additional file 2: Figure S2D and S2E). For example, Micro-C 3 billion data called 2.8 times more loops that are > 1 Mb in distance than Hi-C 1 billion data at 5 kb resolution.

Next, we compared chromatin loops found among Hi-C 1 billion, Micro-C 1 billion, 2 billion, and 3 billion data to see how many of these loops were shared among each other at 5 kb resolution. Most of chromatin loops found in each dataset were also found in Micro-C 2 billion or Micro-C 3 billion data, with Micro-C 3 billion data identifying > 68% more unique loops than the others (Fig. 2D, Additional file 2: Figure S2F and S2G). When we further examined the distance of unique and shared loops, we found that unique loops found in Micro-C 2 billion data and Micro-C 3 billion data had longer distance than the shared loops (Additional file 2: Figure S2H-S2K). Particularly, Micro-C 3 billion data detected a lot of additional > 1 Mb-sized loops that were not detected from other datasets. Similar patterns are found in both 10 kb resolution and 5 kb resolution analyses, indicating that deeply sequenced Micro-C data outperforms less sequenced data on identifying long-range loops.

### Structural variants and interchromosomal loops in prostate cancer cells are identified using Micro-C

**Fig. 3:**
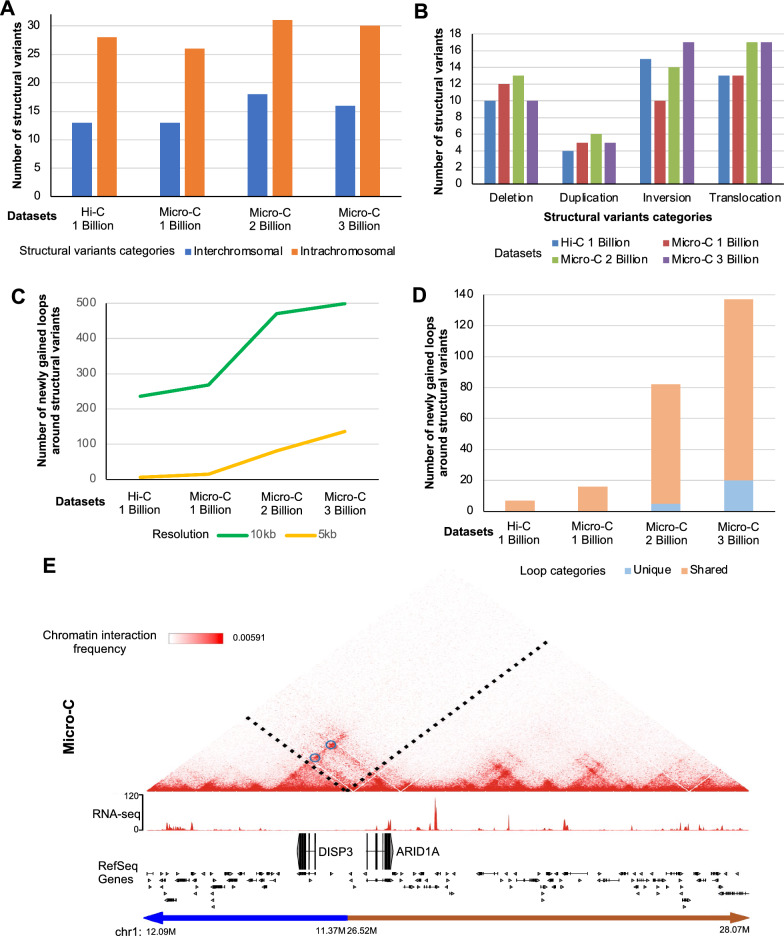
Chromatin loops near structural variants. **A** Numbers of inter- and intra-chromosomal structural variants identified from Hi-C and Micro-C data are shown. **B** Numbers of each category of structural variants identified from Hi-C and Micro-C data are shown. **C** Numbers of loops identified around the structural variants from Hi-C and Micro-C data are shown at 5 kb and 10 kb resolutions. **D** Numbers of neoloops (loops newly gained due to the structural variants) that are shared (between any datasets) or unique among Hi-C 1 billion, Micro-C 1 billion, 2 billion and 3 billion data are shown. **E** An example heatmap of Micro-C data near the *ARID1A* gene that includes inversion structural variant is shown on the top. Under the heatmap, RNA-seq and RefSeq gene tracks are shown. Example neoloops newly gained due to the structural variants are circled in blue

Genomic rearrangements such as inversions, deletions, and translocations are observed in prostate cancer cells [22–25]. Structural variants, which are genomic rearrangements that affect large fragments of DNA, are commonly found in cancer genomes and play a key role in tumorigenesis [26]. Previous studies showed that it is possible to identify interchromosomal (between different chromosomes) and intrachromosomal (within a chromosome) structural variants, using chromatin interaction data such as Hi-C [27, 28]. Therefore, we used our C42B prostate cancer Hi-C and Micro-C data to identify structural variants using NeoLoopFinder [27]. We identified 13–18 interchromosomal structural variants and 26–31 intrachromosomal structural variants from Hi-C and Micro-C data. In total, 41 structural variants were found in Hi-C 1 billion data, 39 in Micro-C 1 billion data, 47 in Micro-C 2 billion data, and 46 in Micro-C 3 billion data (Fig. 3A, Additional file 4: Table S3).

When we compared the identified structural variants, there were no big differences in the number of deletions, duplications, inversions, or translocations identified among datasets (Fig. 3B). However, the number of chromatin loops newly gained due to structural variants (neoloops) increased as the read number increased (Fig. 3C). At 5 kb resolution, we identified 6 loops in Hi-C 1 billion data, 15 in Micro-C 1 billion data, 81 in Micro-C 2 billion data, and 136 in Micro-C 3 billion data (Fig. 3C). When we compared the newly gained loops around structural variants from datasets, the loops were largely shared among datasets, but the greatest number of loops was identified from Micro-C 3 billion data only (unique loops) (Fig. 3D). For example, we identified new chromatin interactions that were induced by inversion at chromosome 1p36 region. Inversion of chromosome 1p36 region established new loops between 11.5Mbp and 27Mbp region near the *ARID1A* and *DISP3* genes (Fig. 3E). By overlaying Micro-C signals with RNA-seq signals, we noted that the *ARID1A* gene, which was reported to be dysregulated in prostate tumors [29], was lowly expressed in C42B prostate cancer cells while the *DISP3* gene was not expressed.

### A subset of regulatory elements is involved in chromatin loops

**Fig. 4:**
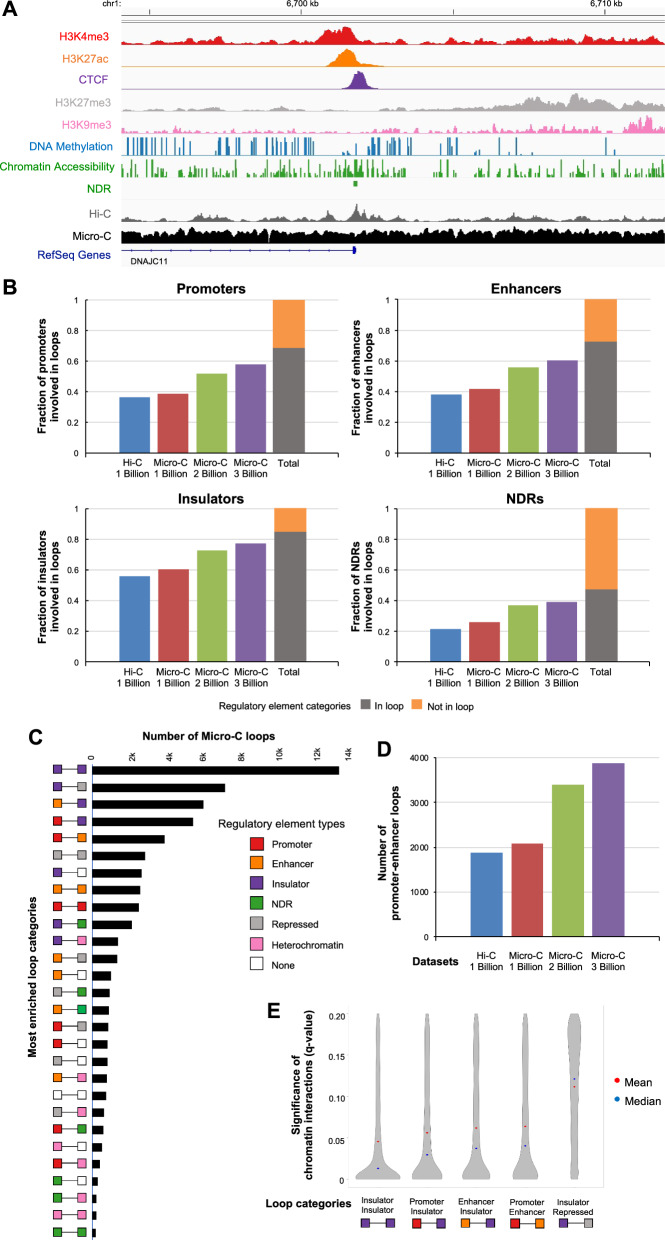
Regulatory elements and nucleosome-depleted regions (NDRs) that are involved in loops. **A** Genome browser screenshots of ChIP-seq (H3K4me3, H3K27ac, CTCF, H3K27me3, H3K9me3), NOMe-seq (DNA methylation, chromatin accessibility, nucleosome-depleted regions (NDRs)), Hi-C, Micro-C, and RefSeq Genes are shown. **B** Fractions of regulatory elements that intersect with loop anchors identified from Hi-C 1 billion, Micro-C 1 billion, 2 billion, and 3 billion data are plotted (left). A fraction of regulatory elements that intersect with loop anchors from any datasets is shown in grey (in loop) while the one not in loop is shown in orange (not in loop) (right). **C** Numbers of loops belong to different loop categories defined by intersecting the loop anchors with different types of regulatory elements (red: active promoter, orange: active enhancer, purple: active insulator, green: NDRs without features, grey: repressed region, pink: heterochromatin region, and white: none) are shown. They are in rank order with the most frequent category at the top and the 28th most frequent category at the bottom. Chromatin loops are called at 5 kb resolution using Micro-C 3 billion data. **D** Comparison of number of promoter–enhancer loops identified from Hi-C 1 billion, Micro-C 1 billion, 2 billion and 3 billion data. **E** Significance of chromatin interaction (*q*-value identified by Mustache) for top 5 loop categories. A mean *q*-value is shown in red. A median *q*-value is shown in blue

Regulatory elements are reported to be involved in looping [21]. Therefore, we further examined the regulatory elements that were involved in chromatin looping. To identify active regulatory elements, we used ChIP-seq using specific antibodies of H3K4me3 (*n* = 12,716), H3K27ac (*n* = 30,329), and CTCF (*n* = 38,130), identifying reproducible and robust peaks from ChIP-seq replicates following the ENCODE guideline [30]. We also used H3K27me3 and H3K9me3 ChIP-seq to identify reproducible repressed regions (*n* = 379,103) and heterochromatin regions (*n* = 140,678), respectively. Furthermore, to identify NDRs (*n* = 65,838) and nucleosome positions at single molecule resolution, we used NOMe-seq (Fig. 4A, Additional file 1: Table S1).

We next calculated the number of active promoters (defined using 2 kb windows of transcription start sites (TSSs) of expressed genes from RNA-seq, *n* = 27,002), active enhancers (defined as > 2 kb of TSSs with H3K27ac ChIP-seq peaks, *n* = 22,653), active insulators (defined as > 2 kb of TSSs with CTCF ChIP-seq peaks not found in active enhancers, *n* = 15,346), and NDRs without features (NDRs identified by NOMe-seq not found in active promoters, enhancers, and insulators, *n* = 28,870) that are involved in loops from Hi-C and Micro-C data. We and others previously found that the number of regulatory elements involved in looping from Hi-C is relatively small to the total number of regulatory elements [31, 32]. When we performed integrative analysis using Hi-C 1 billion, Micro-C 1 billion, 2 billion, and 3 billion data, we found that less than 40% of promoters were located at chromatin loop anchors of Hi-C 1 billion data and Micro-C 1 billion data, and 57% of promoters were found for Micro-C 3 billion data at 5 kb resolution (Fig. 4B). As different chromatin loops can be called by each data, we determined the total number of promoters involved in looping (promoters that intersected with loop anchors). Interestingly, even when we combined all promoters involved in loops from all datasets, only 69% of promoters were involved in chromatin looping. When we performed analyses for enhancers, similar patterns were detected (Fig. 4B). Insulators had the largest percentage of regions intersected with loop anchors as previous studies have shown that insulators are more enriched at loop anchors compared to promoters and enhancers [21, 31, 32]. However, even from combined data, we found that 85% of insulators were involved in chromatin looping (Fig. 4B). For NDRs that do not overlap with promoters, enhancers, and insulators (NDRs without features), we found that 54% of them were located at chromatin loop anchors (Fig. 4B). Our findings suggest that a subset of active regulatory elements is involved in chromatin looping.

Next, we determined loop categories by overlapping each chromatin loop anchor with promoters, enhancers, insulators, other NDRs, repressed regions, and heterochromatin regions (Fig. 4C). Looking at the loop categories, we found that the most common form of loop category was insulator–insulator, as expected from the high percentage of insulators intersecting with loops and previous studies reported [21, 33, 34], followed by insulator–repressed, and insulator–enhancer loop categories (Fig. 4C). The promoter–enhancer loop category was the fifth most common loop category even when we gave more priority on defining regulatory elements (see Methods) in Micro-C 3 billion data (Fig. 4C). When we performed analysis using different resolutions and Hi-C data and smaller reads Micro-C data, similar loop categories and ranks were observed, including the analysis which was performed at 1 kb resolution (Additional file 2: Figure S4).

The promoter–enhancer loop category is seen as an underlying transcription regulation by bringing an enhancer to interact with a promoter to regulate gene expression [35]. When we compared Hi-C 1 billion, Micro-C 1 billion, 2 billion, and 3 billion data, the number of promoter–enhancer loops identified slightly increased from Hi-C 1 billion data to Micro-C 1 billion data and saw bigger increases at Micro-C 2 billion and 3 billion data (Fig. 4D). However, the total number of promoter–enhancer loops was small compared to the total number of active promoters and enhancers, supporting that only a subset of promoters and enhancers is involved in chromatin loops. Comparison of statistical significance of chromatin interactions of the top 5 loop categories revealed that there were no differences in the distribution of *q*-values for insulator–insulator, promoter–insulator, enhancer–insulator, and promoter–enhancer loops except for insulator–repressed loops (Fig. 4E). The insulator–repressed loops had slightly higher *q*-value (indicating less significant and lower chromatin interaction counts) than insulator–insulator, promoter–enhancer, enhancer–insulator, and insulator–insulator loops (Fig. 4E, Additional file 2: Figure S5A).

Moreover, we compared the gene expression level of active promoters involved in different loop categories. We detected no noticeable differences in gene expression among active promoters involved in most loop categories (Additional file 2: Figure S5B). However, we found that gene expression level of promoters involved in promoter–heterochromatin loops had lower expression levels than promoters involved in promoter–enhancer loops and promoter–insulator loops (*p*-value < 3.14 e−5, *p*-value < 1.18 e−4, respectively) (Additional file 2: Figure S5B). When we also tested if ChIP-seq signals or NOMe-seq signals differ between regulatory elements that belong to different loop categories, we identified some marginal differences (Additional file 2: Figure S5C-H). For example, H3K27ac ChIP-seq signal values were relatively stronger for ones involved in enhancer–promoter loops than enhancer–repressed loops and enhancer–heterochromatin loops (*p*-value < 4.18e−15, *p*-value < 1.76 e−05, respectively) (Additional file 2: Figure S5D).

### Promoter capture Micro-C identifies additional chromatin interactions involved in promoters which are not detected by Micro-C

**Fig. 5:**
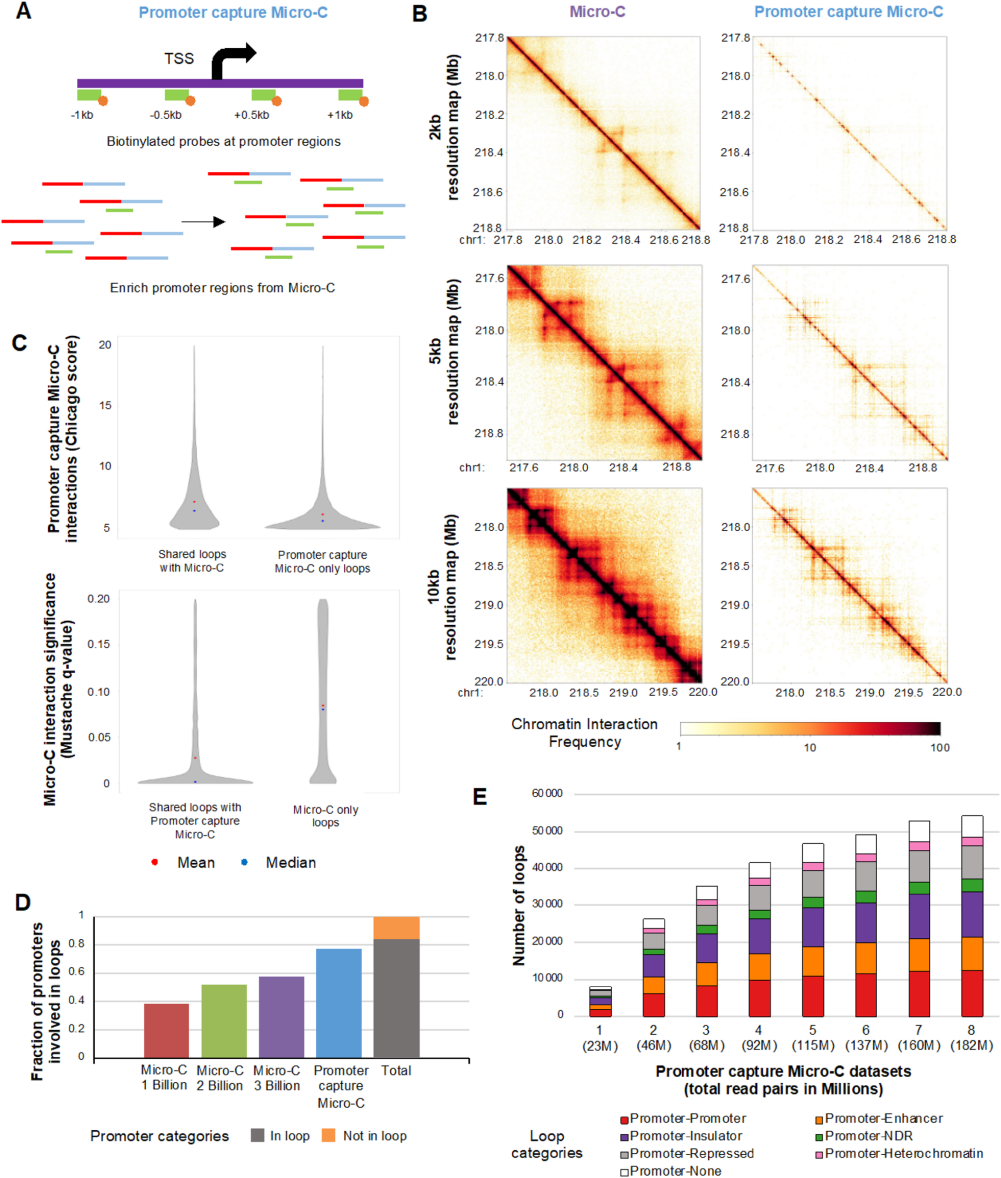
Promoter capture Micro-C data analysis. **A** An overview of promoter capture Micro-C experimental procedure, including the promoter probe design scheme. Probes (green bar) with biotins (orange circle) are designed surrounding TSSs, and Micro-C reads are pulled down using the probes for promoter capture Micro-C. **B** Chromatin interaction heatmaps of Micro-C and promoter capture Micro-C data near chr1q41 region at 2 kb (top), 5 kb (middle), and 10 kb (bottom) resolutions. **C** Significance of chromatin interaction (Chicago score (-log *p*-value), Mustache (*q*-value)) for loops found in both promoter capture Micro-C and Micro-C (shared) and only one data is plotted. A mean value in shown in red. A median value is shown in blue. **D** Fractions of active promoters that intersect with the loop anchors from Micro-C 1 billion, 2 billion, 3 billion data or promoter capture Micro-C data are shown (left). A fraction of active promoters that intersect with loop anchors from any datasets is shown in grey (in loop) while the one not in loop is shown in orange (not in loop) (right). **E** Numbers of promoter-involved loops and loop categories (red: active promoter–active promoter, orange: active promoter–active enhancer, purple: active promoter–active insulator, green: active promoter–NDRs, grey: active promoter–repressed region, pink: active promoter–heterochromatin region, and white: active promoter–none) identified from promoter capture Micro-C data are shown. Loops are called at 5 kb resolution

While Micro-C 3 billion data identified numerous chromatin loops, we found that only the subset of promoters was identified to be involved in loops, and the number of promoter–enhancer loops identified was still relatively low compared to insulators-involved loops (Fig. 4C). This could be due to the fact that insulators-involved loops are dominant for genome-wide Micro-C reads. Therefore, we tested by performing promoter capture Micro-C with probes that are designed to capture promoter-specific chromatin interactions from Micro-C (Fig. 5A, Additional file 2: Figure S6A). Probes (120 bp in size) were designed to − 1 kb, − 0.5 kb, + 0.5 kb, + 1 kb of total 315,286 TSSs and were used to pull down promoter regions from 8 Micro-C replicates by generating 8 promoter capture Micro-C libraries; each library was sequenced about 20 million read pairs (Additional file 1: Table S1). 161,144 probes that span total 19,337,280 base pairs were used. There were total of 68,206,114 valid read pairs after filtering duplicates and invalid read pairs, which indicates that there were 423 valid read pairs per probe (Additional file 1: Table S1).

To compare chromatin interactions between Micro-C data (total 3 billion read pairs) and promoter capture Micro-C data (total 182 million read pairs), we generated heatmaps at 2 kb, 5 kb, and 10 kb resolutions (Fig. 5B). Promoter capture Micro-C heatmaps at higher resolutions such as 2 kb and 5 kb resolutions did not display similar patterns as Micro-C heatmaps because reads are sparse and specific regions are enriched for promoter capture Micro-C data (Fig. 5B). When we called TADs using promoter capture Micro-C data with TopDom program [36], we identified 5,535 TADs (Additional file 3: Table S2). When we compared these identified TADs with the TADs identified from Micro-C 3 billion data, 76% of them were found common, which indicated that a large portion of TADs overlapped to each other, but the percentage of overlap was relatively less compared to the ones we calculated among Micro-C 1 billion, 2 billion, and 3 billion data (Additional file 3: Table S2).

To investigate chromatin loops of promoter capture Micro-C data, we called chromatin loops from promoter capture Micro-C datasets using Chicago loop calling program [37]. When we measured the number of chromatin loops from promoter capture Micro-C by increasing the number of libraries and reads of sequenced, we found that the number of identified loops continued to increase but started to plateau around 160 million read pairs (Additional file 2: Figure S6B). We identified 10,000 to over 70,000 loops from 20 million read pairs to 180 million read pairs datasets (Additional file 2: Figure S6B, Additional file 4: Table S3). We were able to identify 73,833 chromatin loops at 5 kb resolution using the promoter capture Micro-C data that includes 182 million read pairs (Additional file 4: Table S3). When we compared the identified chromatin loops with Micro-C data, the loops that were identified in both promoter capture Micro-C data and Micro-C 3 billion data had significantly more chromatin interaction counts reflected with higher Chicago scores and lower Mustache *q*-values than loops that were only found in each data although all of loops were still comparably enriched (Fig. 5C, Mustache *q*-value < 0.20, Chicago score > 5). When we further compared virtual 4C profiles of promoter capture Micro-C and Micro-C data using 3D genome browser [38], we were able to see that interaction patterns are consistent between datasets (Additional file 2: Figure S7), but the quality of overall interaction maps of deeply sequenced Micro-C data was higher than promoter capture Micro-C data.

To determine how many promoters were involved in loops, detected from promoter capture Micro-C, we calculated the percentage of promoters involved in looping. We found that more promoters were intersected with anchors of the promoter capture Micro-C identified loops, compared to Micro-C 3 billion data (Fig. 5D). However, there were still 15% of promoters which were not involved in looping. When we intersected the other side of the identified loop anchor of promoter capture Micro-C loops with other active regulatory elements, most of the promoters were either looped to promoters, insulators, enhancers, or repressed regions (Fig. 5E). Significance of promoter capture Micro-C chromatin loops measured by Chicago scores among loop categories showed similar levels except that promoter–heterochromatin loops had lower Chicago scores (less interaction counts) than other loop categories (Additional file 2: Figure S6C). In summary, promoter capture Micro-C identified additional loops involving promoters and other regulatory elements while utilizing a lower amount of sequencing reads than Micro-C.

### More highly phased nucleosomes are observed surrounding the active regulatory elements involved in chromatin loops

**Fig. 6:**
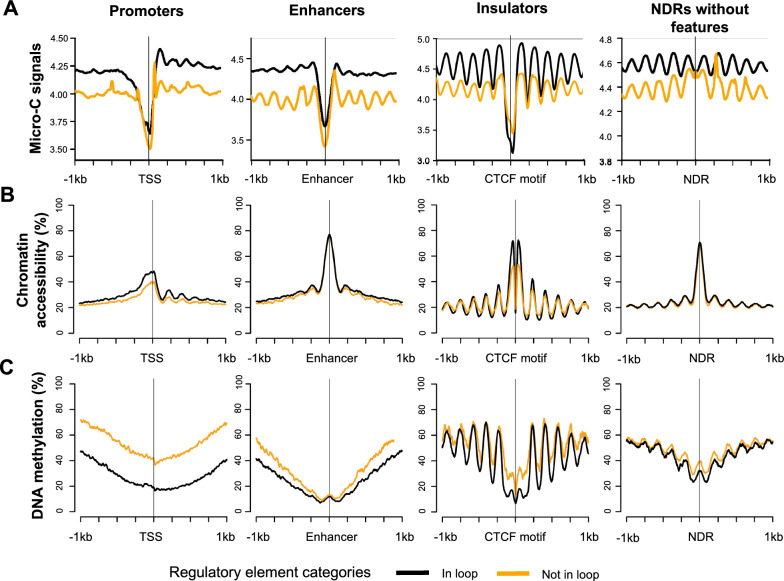
Nucleosome phasing and DNA methylation levels around regulatory elements involved in loops. **A** Average Micro-C signals around active promoters, enhancers, insulators, and NDRs without features (NDRs that do not overlap with active promoters, enhancers, and insulators) that are in loop (black) vs those that are not in loop (orange) are shown. **B** Average chromatin accessibility levels (%) of active promoters, enhancers, insulators, and NDRs without features that are in loop (black) vs those that are not in loop (orange) are shown. **C** Average DNA methylation levels of active promoters, enhancers, insulators, and NDRs without features that are in loop (black) vs those that are not in loop (orange) are shown

Next, we visualized Micro-C MNase signals at regulatory elements using Micro-C 3 billion data. At active promoters, we found a substantial dip near TSSs with nucleosome phasing at downstream regions, indicating that they are largely accessible (Fig. 6A). A dip of Micro-C signals and surrounding nucleosome phasing patterns were also detected in other regulatory elements, such as active enhancers, active insulators, and NDRs without features. When we categorized active regulatory elements into two groups: ones that are enriched at chromatin loop anchors (in loop) and ones that are not enriched at chromatin loop anchors (not in loop), we found that nucleosome phasing signals surrounding the regions were different (Fig. 6A). For example, stronger dips and highly phased nucleosome signals were detected at active promoters and insulators involved in loops compared to ones that were not involved in loops (*p*-value < 0.023).

We further compared nucleosome positioning of active regulatory elements involved in loops and not in loops using independent NOMe-seq data (Fig. 6B). NOMe-seq method is based on the treatment of chromatin with the M.CviPI methyltransferase, which identifies chromatin accessible regions by methylating GpC dinucleotides that are not protected by nucleosomes at single molecule resolution. NOMe-seq also maps endogenous CpG DNA methylation patterns genome-wide [14, 15]. Like Micro-C MNase signals showed, we found more accessible regions at active promoters and insulators involved in loops than ones not involved in loops (*p*-value < 2.20 e−16). DNA methylation levels were relatively lower in the promoters and insulators involved in loops than ones not involved in loops (*p*-value < 2.20 e−16) (Fig. 6C). This observation was still detected when we randomly selected the same number of regulatory elements per category (in loop vs not in loop) and visualized the Micro-C and NOMe-seq signals (Additional file 2: Figure S8A–C).

H3K4me3 ChIP-seq signals were also stronger for active promoters in loops compared to those not in loops, and the similar pattern was also observed in CTCF ChIP-seq signals (Additional file 2: Figure S8D). ChIP-seq signal difference was consistent even when we randomized promoters and insulators and tested (Additional file 2: Figure S8E). Next, we checked if gene expression level was different between active promoters that are located at loop anchors and the ones that are not. We found that gene expression levels of active promoters involved in loops were not significantly different than those not found in loops, on average (*p*-value = 0.116) (Additional file 2: Figure S8F). Promoters that were found to be in promoter capture Micro-C loop anchors also had higher ChIP-seq signals and significantly higher accessibility level than the promoters that did not intersect with promoter capture Micro-C loop anchors (Additional file 2: Figure S6D and E, *p*-value < 2.20e−16). This finding summarizes that among active promoters and insulators, ones enriched at chromatin loop anchors are more accessible, nucleosome depleted, and surrounded with stronger nucleosome positioning than those not found at chromatin loop anchors.

## Discussion

Previous studies reported that Micro-C can identify chromatin interactions at high resolution [9, 16, 17]. However, it was not clear how many sequencing reads are needed to detect chromatin loops at what resolutions in human cancer cells. To investigate that, we performed Hi-C, Micro-C, and promoter capture Micro-C in C42B human prostate cancer cells and compared the coverage by changing the sequencing reads. When we compared Hi-C 1 billion data and Micro-C 1 billion data of human prostate cancer cells, we found that Micro-C identifies > 23% more chromatin loops than Hi-C (Fig. 1). Deep sequencing of Micro-C data revealed that more than 2 billion read pairs of Micro-C are needed to capture chromatin interactions at 1 kb resolution. Interestingly, loops newly identified from > 2 billion Micro-C data were longer in size than loops (> 1 Mb) found from 1 billion data (Fig. 2). We also found that increasing the sequencing depth of Micro-C allowed to identify more newly gained loops around structural variants (Fig. 3). Unlike chromatin loops called at high resolution, we did not see that much difference on calling TADs among Hi-C 1 billion data and differently sequenced Micro-C data (Fig. 1, Additional file 3: Table S2). This was due to the fact that TADs can be pretty accurately identified using low-resolution chromatin interaction data (e.g., 50 kb resolution).

By integrating ChIP-seq and NOMe-seq data with Micro-C data, we characterized regulatory elements and NDRs that were involved in chromatin loops. In accordance with previous findings [21, 33, 34], insulator–insulator loops were most frequently found from our Micro-C data (Fig. 5). Increasing the sequencing depth of Micro-C allowed to detect more promoter–enhancer loops, but the number of identified promoter–enhancer loops from deep-sequencing Micro-C data was still relatively low (Fig. 4). To capture more chromatin interactions, we performed promoter capture Micro-C, which identified additional chromatin loops involved in promoters that were not detected by Micro-C. Sequencing of 160 million read pairs of promoter capture Micro-C data resulted in reaching a plateau of identifying chromatin loops that promoters were involved in (Fig. 5, Additional file 2: Figure S6B). Although overall quality and resolution of promoter capture Micro-C interaction map was lower than deeply sequenced Micro-C data, this finding indicates that promoter capture Micro-C method can detect chromatin interactions of promoter regions identified from Micro-C in an efficient manner with less sequencing (Fig. 5, Additional file 2: Figure S7). Previous capture Micro-C and MNase 3C capture studies [39–41] were focused on targeted regions and may provide higher sensitive signals using a relatively small number of reads. However, these methods detect limited chromatin interactions at the target regions while promoter capture Micro-C detects chromatin interactions of all promoter regions throughout the genome.

Interestingly, when we used promoter capture Micro-C, we found that > 15% of promoters were still not involved in loops called from deeply sequenced promoter capture Micro-C data (Fig. 5). Moreover, the total number of promoter–enhancer loops identified was less than 5,000. These findings could indicate that promoter–enhancer interaction is too dynamic to capture using 3C-based assays, or only a subset of promoters physically interact with enhancers. Furthermore, the number of functional enhancers that interact with promoters could be a lot less than what we estimated based on H3K27ac ChIP-seq. A previous study indicated that not all of H3K27ac marked enhancers are functional [42].

When we compared the characteristics of regulatory elements and NDRs that were involved in chromatin loops against those that were not, interestingly, the promoters and insulators located at the loop anchors had more highly phased nucleosomes and stronger nucleosome positioning than the ones not located at loop anchors (Fig. 6). Moreover, chromatin accessibility level was higher while DNA methylation level was lower for these regulatory elements located at the loop anchors. Particularly, promoters had substantial differences in DNA methylation and chromatin accessibility levels compared to other regulatory elements. This finding supports the idea that chromatin interaction is related to nucleosome positioning [43]. Several studies have reported the importance of nucleosome positioning in regulating gene expression [44, 45]. Although we saw differences in nucleosome phasing levels between active promoters located at loop anchors and ones that are not in loops, gene expression levels of active promoters were not significantly different regardless of looping (Additional file 2: Figure S8F). When we further measured gene expression levels of active promoters belonging to different loop categories, we found that active promoters, which were looped to inactive regulatory elements (i.e., heterochromatin regions marked with H3K9me3, repressed regions marked with H3K27me3), had slightly lower expression than active promoters looped to active regulatory elements from Micro-C data analysis (Additional file 2: Figure S5B). Phanstiel et al. previously reported that not only active promoters of genes that are highly expressed, but also inactive promoters are often involved in looping [46]. Based on this, it is suggested that the promoter involvement of chromatin looping does not always increase the gene expression levels. Moreover, gene expression levels of the active promoters involved in loops appeared to be affected by the characteristics of regulatory elements located at the other anchor, but further investigation is needed to better understand the chromatin structure of gene regulation.

## Conclusions

In conclusion, we performed Hi-C, Micro-C, and promoter capture Micro-C in prostate cancer cells and assessed to determine the required library and read numbers to generate high-resolution three-dimensional (3D) chromatin interaction maps and loops. The number of identified promoter–enhancer loops increased by increasing the sequencing depth of promoter capture Micro-C. However, the number of promoter–enhancer loops identified from deep-sequencing data was still relatively small, compared to the total number of enhancers identified from H3K27ac ChIP-seq. By investigating the relevance of nucleosome positioning and chromatin interactions, we observed the possible effect of chromatin interactions in DNA methylation and nucleosome phasing. Our findings also suggest the presence of distinct promoter groups, which are differently involved in chromatin structures and gene regulation. This work will benefit research community by providing a framework and guidelines for designing research projects on chromatin interactions among regulatory elements and NDRs.

## Methods

### Cell culture

The human prostate cancer C42B cells were obtained from ATCC (Cat # CRL-3315, ATCC, Manassas, VA, USA). Cells were grown at 37 °C in 5% CO2. It was grown in RPMI1640 culture medium and supplemented with 10% fetal bovine serum (Gibco by Thermo Fisher Scientific, Waltham, MA, USA) and 1% penicillin and streptomycin. All cell stocks were authenticated at the USC Norris Cancer Center cell culture facility by comparison to the ATCC and/or published genomic criteria for that specific cell line; all cells were documented as free of mycoplasma.

### In situ Hi-C

Hi-C fastq files were obtained from previous experiments, which were performed in-house (GSE118629) [31]. In Situ Hi-C experiments followed the original protocol by Rao et al. with minor modifications. MboI restriction enzyme was used for digestion, and T4 DNA Ligase (Cat # M0202, New England Bio Labs, Ipswich, MA, USA) was used for ligation. Chromatin was sheared to 300–500 bp with Covaris instrumented, and biotin-tagged DNA was pulled down with Dynabeads MyOne Streptavidin C1 beads (Cat # 65,002, Life technologies, Carlsbad, CA, USA) with 2 × Binding Buffer (2 × BB: 10 mM Tris–HCl (pH 7.5), 1 nM EDTA, 2 M NaCl). Hi-C libraries were amplified and sequenced in Illumina HiSeq 2000.

### Micro-C

1 × 10^6^ cells were harvested and cross-linked with DSG and formaldehyde at room temperature. Cells were digested with MNase Enzyme Mix (Cat # PN DG-NUC-001, Dovetail Genomics, Scotts Valley, CA, USA) and were lysed with SDS. Lysate was mixed with Chromatin Capture Beads (Cat # PN DG-REF-001, Dovetail Genomics, Scotts Valley, CA, USA) to bind chromatin and incubated at room temperature for 10 min. End Polishing Master Mix was added to lysate and incubated for 30 min at 22 °C and 30 min at 65 °C to end polish (Cat # PN DG-NUC-001, Dovetail Genomics, Scotts Valley, CA, USA). For ligation, Bridge Ligation Mix (Cat # PN DG-NUC-001, Dovetail Genomics, Scotts Valley, CA, USA) and Bridge Ligase (Cat # PN DG-NUC-001, Dovetail Genomics, Scotts Valley, CA, USA) were added and incubated at 22 °C for 30 min, and Intra-aggregate Ligation Buffer (Cat # PN DG-NUC-001, Dovetail Genomics, Scotts Valley, CA, USA) and Intra-aggregate Ligation Enzyme Mix (Cat # PN DG-NUC-001, Dovetail Genomics, Scotts Valley, CA, USA) were added and incubated at 22 °C for 1 h. DNA were isolated by reverse-crosslinking with Proteinase K and Crosslink Reversal Buffer, and incubating at 55 °C for 15 min, 68 °C for 45 min. DNA was then purified. For library preparation, purified DNA was added with End Repair Master Mix (Cat # PN DG-LIB-001, Dovetail Genomics, Scotts Valley, CA, USA) and incubated at 20 °C for 30 min and 65 °C for 30 min for end repair. Adaptor for Illumina, ligation enzyme mix and ligation enhancer were added for adapter ligation, then DNA was purified using SPRIselect beads (Cat # CNGS050, Bulldog Bio Inc, Portsmouth, NH, USA). Adaptor-ligated DNA was then washed and captured with Streptadvidin beads (Cat # PN DG-REF-001, Dovetail Genomics, Scotts Valley, CA, USA). PCR was performed, and size range between 350–1,000 bp was selected for the library. Prepared libraries were then sequenced with Illumina sequencers.

### Promoter capture Micro-C

For Promoter capture Micro-C, Micro-C libraries (total 8 libraries) prepared using the above procedures were used. First, each library was pooled for hybridization reaction. Probe solution, made up of hybridization mix (Cat # CP2-HM-001, Dovetail Genomics, Scotts Valley, CA, USA), pan promoter panel (Cat # CP3-PP-001, Dovetail Genomics, Scotts Valley, CA, USA) and water, was prepared and used for each hybridization reaction. For hybridization reaction, the probe solution was first heated to 95 °C for 2 min in a thermal cycler with a lid at 105 °C. Library was heated in similar way for 5 min. After cooling in ice for 5 min, and equilibrating to room temperature for 5 min, the probe solution was added to the library. Hybridization Enhancer (Cat # CP2-HM-001, Dovetail Genomics, Scotts Valley, CA, USA) was then added and the mixed solution was incubated at 70° for 16 h in a thermal cycler with the lid at 85 °C. After hybridization steps, the hybridized targets were washed and captured with Streptavidin beads (Cat # CP1-EM-001, Dovetail Genomics, Scotts Valley, CA, USA), amplified with PCR, and then purified. Prepared libraries were sequenced with Illumina sequencers.

### Chromatin interaction data processing

Hi-C 1 billion data includes total 1,094,888,777 raw read pairs, Micro-C 1 billion data includes total 1,050,616,368 raw read pairs, Micro-C 2 billion includes total 2,335,898,791 raw read pairs, and Micro-C 3 billion data includes total 3,430,994,736 raw read pairs (Additional file 1: Table S1). Both Micro-C and Hi-C data were processed using the 4DN Data Portal’s Hi-C processing pipeline (https://data.4dnucleome.org/resources/data-analysis/hi_c-processing-pipeline). Raw sequencing reads (fastq files) were first aligned to genome (hg38) using BWA MEM [18], and the aligned reads were paired, sorted, and filtered for PCR duplicates and invalid pairs using Pairtools (https://pairtools.readthedocs.io/en/latest) and converted into pairs files. Resulting pairs files were used to normalize and generate matrix files with Juicer [47]. When generating matrix files from Hi-C data, restriction enzyme information was incorporated, but not with Micro-C data since it did not use restriction enzymes. Matrix files were generated at different resolutions and used for downstream analysis for Juicer [47] (hic files), Cooler [48] (cool files), and Samtools [49] (paired bam files). Bigwig files were generated from paired bam files with the bamCoverage function (normalized using RPKM) of DeepTools [50]. Promoter capture Micro-C data were processed in the same manner.

### TAD identification

First, hic files, which were generated using unique valid read pairs, were converted into sparse format with straw python package (https://github.com/igvteam/hic-straw). Sparse format was then converted into dense format using HiCcompare R package [51] and processed to call TADs using TopDom [36]. To identify TADs, matrix files at 50 kb resolution were used with window size set up as 5. The genomic coordinates (hg38) of identified TADs are listed in Additional file 3: Table S2.

### Chromatin loop identification

Chromatin loops were identified using Mustache [19], HiCCUPS [21], and SIP [20] loop calling programs at 50 kb, 25 kb, 10 kb, 5 kb, 2 kb and 1 kb resolutions with hic files, which were generated using unique valid read pairs,. These programs were selected because they did not require restriction enzyme information to be run, allowing to call loops from Micro-C data. To identify chromatin loops whose anchors are intersected with regulatory elements, we used 1 kb and 5 kb resolution chromatin interaction matrices of Micro-C and Hi-C datasets. To identify chromatin loops from promoter capture Micro-C data, we used Chicago loop calling program [37]. Because the current version of Chicago requires restriction enzyme information, which we do not have for promoter capture Micro-C data, we run the Dovetail Genomics script, which produces the restriction enzyme map for Micro-C data that cuts uniformly throughout the genome (https://github.com/dovetail-genomics/capture/tree/main/docs/source) before calling loops using Chicago.

### ChIP-seq

All ChIP-seq experiments were performed using H3K4me3 antibody (Cat # 9751S.

Cell Signaling and Technology, Inc., Danvers, MA, USA), H3K27ac antibody (Cat # 39,133, Active Motif, Carlsbad, CA, USA), CTCF antibody (Cat # 61,311, Active Motif, Carlsbad, CA, USA), H3K27me3 antibody (Cat # 9733, Cell Signaling and Technology, Inc., Danvers, MA, USA), and H3K9me3 antibody (Cat # 13,969, Cell Signaling and Technology, Inc., Danvers, MA, USA) as part of previous studies [31, 44], and datasets were validated according to ENCODE standards. H3K27ac (ENCSR279KIX) and CTCF (ENCSR460LGH) ChIP-seq data were obtained from ENCODE. H3K4me3, H3K27me3, and H3K9me3 ChIP-seq data were obtained from previous experiments (GSE102616, GSE40050, and GSE118629) (Additional file 1: Table S1). ChIP-seq data were aligned with BWA using hg38, and filtered with Picard (http://broadinstitute.github.io/picard/) and Samtools [49]. Peaks were then called with MACS2 and were checked for reproducibility with IDR [52] with FDR at 0.05.

### RNA-seq

RNA was extracted with Trizol reagent (Cat # 15,596–018, Thermo Fisher Scientific, NY, USA) then assessed with a 2100 Bioanalyzer instrument (Cat # G2939AA, Agilent Technologies, Santa Clara, CA, USA). RNA-seq libraries were prepared with KAPA Stranded mRNA-Seq Kit with KAPA mRNA Capture Beads (Cat # KK8421, Kapa Biosystems, Woburn, MA, USA) then sequenced on an Illumina NextSeq 500 with 75 bp single end reads. RNA-seq was performed with triplicate. Fastq files of RNA-seq data were obtained from previous experiment (GSE118629) (Additional file 1: Table S1). RNA-seq data were first trimmed with Trimgalore (https://github.com/FelixKrueger/TrimGalore), and aligned with STAR [53] using hg38. It was then quantified with Rsem [54] and HTseq [55].

### NOMe-seq

NOMe-seq data were generated in C42B prostate cancer cells as part of the previous study (GSE102616) [44] (Additional file 1: Table S1). First, chromatin was treated with M.CviPI methyltransferase (Cat # M0227B, New England Bio Labs, Ipswich, MA, USA) to methylate GpC dinucleotides and was followed by bisulfite treatment to methylate CpG dinucleotides, then sequenced with Illumina HiSeq 2000 sequencer. NDRs, identified with C42B NOMe-seq data in hg19 from the previous study [44] were lifted over to hg38 using UCSC genome browser’s liftover tool (https://genome.ucsc.edu/cgi-bin/hgLiftOver).

### Characterization of loops

Active promoters were defined as ± 2 kb windows from TSS of transcripts had higher than average 0.5 FPKM across the replicates (*n* = 27,004). Active enhancers were defined as > 2 kb of TSSs with H3K27ac ChIP-seq peaks (*n* = 22,653), and active insulators were defined as > 2 kb of TSSs with CTCF ChIP-seq peaks not found in active enhancers (*n* = 15,346) using bedtools2 [56]. H3K27me3 ChIP-seq peaks that were not overlapped with active regulatory elements were defined as repressed regions (*n* = 371,614). H3K9me3 ChIP-seq peaks that were not overlapped with either active regulatory elements or repressed regions were defined as heterochromatin regions (*n* = 135,991) (Additional file 5: Table S4). These regulatory elements were then intersected using fuzzyjoin R package (https://github.com/dgrtwo/fuzzyjoin). Difference_semi_join function of fuzzyjoin R package was used to overlap the genomic locations of regulatory elements and chromatin loop anchors. For 1 kb resolution data, ± 2 kb windows were used, and for 5 kb resolution data, ± 10 kb windows were used to account for chromatin interactions that may not be in the exact bin. Additional file 5: Table S4 lists the genomic coordinates of regulatory elements as well as its overlap status with chromatin loop anchor; score of 1 is given when the regulatory element is intersected with the loop anchor while 0 is given when it did not intersect. When intersecting regulatory elements with loop anchors for Figs. 4, 5, and Additional file 2: Figure S4, regulatory elements were prioritized in following order: promoter–enhancer–insulator–NDRs without features–repressed regions–heterochromatin regions–none.

### Hi-C, Micro-C, and promoter capture Micro-C data visualization

Hi-C, Micro-C, and promoter capture Micro-C chromatin interaction heatmaps were visualized using cooltools (https://github.com/open2c/cooltools) at 10 kb, 8 kb, 5 kb, 4 kb, 2 kb and 1 kb resolutions. Heatmaps were visualized at log scale with max score of 100 to allow comparisons between resolutions and datasets. To visualize signals in Fig. 6, Additional file 2: Figure S6, and Additional file 2: Figure S8, bigwig files generated from Hi-C, Micro-C, and promoter capture Micro-C data were processed with computeMatrix function from DeepTools [50]. The generated matrix files are used to plot signals around regulatory elements with plotHeatmap function from DeepTools [50]. For active promoters, plots were generated at the center of TSSs of active promoters we defined above. For active enhancers, plots were generated at the center of NDRs within active enhancers we defined above. For insulators, plots were generated at the center of CTCF motifs within insulators we defined above. CTCF motifs were downloaded from Homer [57]. For NDRs without features, plots were generated at the center of NDRs that did not intersect with active promoters, active enhancers, and insulators. These regulatory elements were intersected with loop anchors using fuzzyJoin R package (https://github.com/dgrtwo/fuzzyjoin) as above, then separated into the regulatory elements that are in the loops (In loop) versus the regulatory elements that are not in the loops (Not in loop). To adjust the sample size effect, we randomly selected an equal amount of regulatory elements (active promoters: 10,000, active enhancers: 1,000, insulators: 1,000, and NDRs without features: 10,000) that are in loop vs not in loop 10 times using shuf command and generated plots (Additional file 6: Table S5).

### ChIP-seq signal visualization

For Additional file 2: Figure S6 and Additional file 2: Figure S8, ChIP-seq signals of regulatory elements in loop vs not in loop were assessed using bigwig files that were generated by merging ChIP-seq bam files with samtools [49], then converting with Deeptools bamCoverage function [50]. Signals were plotted at the center of above-defined regulatory elements using plotHeatmap function from DeepTools [50]. To adjust the sample size effect, we randomly selected an equal amount of regulatory elements (active promoters: 10,000, active enhancers: 1,000, insulators: 1,000, and NDRs without features: 10,000) that are in loop vs not in loop 10 times using shuf command and generated plots.

### NOMe-seq signal visualization

To visualize DNA methylation and chromatin accessibility levels from NOMe-seq data, Bistools [58] was used with Bigwig files. Signals were plotted at the center of above-defined regulatory elements using plotHeatmap function from DeepTools [50]. Student’s t-test was performed on DNA methylation and chromatin accessibility levels for regulatory elements in loop vs not in loop. To adjust the sample size effect, we randomly selected an equal amount of regulatory elements (active promoters: 10,000, active enhancers: 1,000, insulators: 1,000, and NDRs without features: 10,000) that are in loop vs not in loop 10 times using shuf command and generated plots.

### Shared loop analysis

To calculate how many loops are shared, difference_semi_join from fuzzyJoin (https://github.com/dgrtwo/fuzzyjoin) was used with loops identified from Hi-C, Micro-C 1 billion, 2 billion, 3 billion and promoter capture Micro-C data at 10 kb, 5 kb, 2 kb and 1 kb resolutions (see Characterization of loops section). Because of the nature of difference_semi_join (for 1 kb resolution data, ± 2 kb windows were used, and for 5 kb resolution data, ± 10 kb windows were used to account for chromatin interactions that may not be in the exact bin), there were some cases where one loop from one data intersected with multiple loops in the other data, resulting in unequal amount of loops shared between the datasets. Additional file 4: Table S3 lists the genomic coordinates of loops as well as its overlap status with loops identified from other datasets; score of 1 is given when the loop is shared while 0 is given when the loop was unique.

After identifying the shared and the unique loops in each dataset, cooltools pileup function (https://github.com/open2c/cooltools) was used to analyze average chromatin interactions around the shared and the unique loops. 1 kb bins were used to calculate average interactions, and 100 kb region around the loops were used to view the interactions. For Hi-C 1 billion data and Micro-C 1 billion data comparison, loops generated from 5 kb resolution were used. For Micro-C 3 billion data and promoter capture Micro-C data comparison, loops generated from 5 kb resolution were used.

### Structural variant and neoloop analysis

To identify structural variants in C42B prostate cancer cells with chromatin interaction data, we used hic_breakfinder (https://github.com/dixonlab/hic_breakfinder) and NeoLoopFinder [27]. Cool file matrix files at 25 kb, 10 kb, and 5 kb resolutions were used to calculate, segment, and copy number variations. Structural variants heatmaps, RNA-seq signals, and RefSeq genes were visualized using the visualization tools in NeoLoopFinder python package [27].

### Virtual 4C profiles of Micro-C and promoter capture Micro-C data

To visualize chromatin interaction signals in a 4C-like genome browser, for Micro-C data, we used HiTC [59] to convert the sparse iced matrix files that were generated using HiC-Pro [60] into the dense matrix files. For promoter capture Micro-C data, we used straw [61] to generate the dense matrix files from hic matrix files. Next, we generated btr files from dense matrix files using matrixToButlr.pl script from BUTLRTools (https://github.com/yuelab/BUTLRTools). Then, the btr files were uploaded to 3D genome browser [38] to generate virtual 4C profiles. Both data types were binned at 10 kb resolution.

## Supplementary Information

**Additional file 1: Table S1**. Datasets used in this study. (A) data availability (B) QC statistics of Micro-C, Hi-C, and promoter capture Micro-C data (C) Additional metrics of promoter capture Micro-C data.

**Additional file 2: Figure S1.** Example chromatin interaction heatmaps of Hi-C 1 billion data and Micro-C 1 billion data at high resolutions. (top) 1 kb resolution, (middle) 2 kb resolution, and (bottom) 4 kb resolution. **Figure S2.** Chromatin loops identified using Hi-C and Micro-C data. Numbers of loops identified by (A) SIP and (B) HiCCUPS loop calling programs at different resolutions (50 kb, 25 kb, 10 kb, 5 kb, 2 kb, and 1 kb resolutions) from Hi-C 1 billion, Micro-C 1 billion, Micro-C 2 billion, and Micro-C 3 billion data. (C) Fractions of loops shared among Mustache, SIP, and HiCCUPS loop calling programs. (D) Distribution of the number of loops at different lengths (distances) for Hi-C 1 billion, Micro-C 1 billion, 2 billion and 3 billion data (loops are called at 10 kb resolution). (E) Distribution of the number of loops at different lengths (distances) for Hi-C 1 billion, Micro-C 1 billion, 2 billion and 3 billion data (loops are called at 5 kb resolution). (F) The numbers of loops shared (between any datasets) among Hi-C 1 billion, Micro-C 1 billion, 2 billion, and 3 billion data are plotted. Shared loops were identified with following priority comparisons; Micro-C 3 billion, Micro-C 2 billion, Micro-C 1 billion, Hi-C 1 billion data. (G) An upset plot of Micro-C 3 billion loops showing the number of loops shared with loops identified from different datasets (Hi-C 1 billion, Micro-C 1 billion, Micro-C 2 billion data). (H) Distribution of the number of unique loops at different lengths (distances) for Hi-C 1 billion, Micro-C 1 billion, 2 billion, and 3 billion data (loops are called at 10 kb resolution). (I) Distribution of the number of unique loops at different lengths (distances) for Hi-C 1 billion, Micro-C 1 billion, 2 billion, and 3 billion data (loops are called at 5 kb resolution). (J) Distribution of the number of shared loops at different lengths (distances) for Hi-C 1 billion, Micro-C 1 billion, 2 billion, and 3 billion data (loops are called at 10 kb resolution). (K) Distribution of the number of shared loops at different lengths (distances) for Hi-C 1 billion, Micro-C 1 billion, 2 billion, and 3 billion data (loops are called at 5 kb resolution). **Figure S3.** Example chromatin interaction heatmaps of Micro-C 3 billion data near loops called at 1 kb resolution. (left) shown is a heatmap at chr7:87,200,000–87,700,000 (middle) shown is a heatmap at chrX:9,800,000–10,300,000 (right) shown is a heatmap at chr9:36,000,000–36,500,000. **Figure S4.** The number of loops belong to different loop categories. (A) Hi-C 1 billion data at 5 kb resolution, (B) Micro-C 1 billion data at 5 kb resolution, (C) Micro-C 2 billion data at 5 kb resolution, and (D) Micro-C 3 billion data at 1 kb resolution. They are in rank order with the most frequent category at the top and the 28th most frequent category at the bottom. **Figure S5.** Detailed analysis of chromatin loop categories identified from Hi-C and Micro-C data. (A) Statistical significance of chromatin interactions (*q*-value calculated using Mustache) of Hi-C 1 billion data (left), Micro-C 1 billion data (middle), and Micro-C 2 billion data (right) for top 5 most frequent loop categories. (B) Gene expression levels of active promoters belong to different loop categories. Loops are called using Micro-C 3 billion data at 5 kb resolution. Statistical significance (*p*-value) of gene expression level differences among groups are measured by performing Student’s t-test. Distribution of ChIP-seq signals for (C) active promoter (H3K4me3) loop categories, (D) active enhancer (H3K27ac) loop categories, (E) active insulator (CTCF) loop categories, (F) repressed region (H3K27me3) loop categories, and (G) heterochromatin region (H3K9me3) loop categories. (H) Distribution of chromatin accessibility signals for NDR loop categories. A mean value is shown in red, and median value is shown in blue. **Figure S6.** Detailed analysis of promoter capture Micro-C data. (A) Genome browser screenshots of ChIP-seq, NOMe-seq, promoter capture Micro-C, and Micro-C data near the *STEAP2* gene. (B) Number of loops identified from promoter capture Micro-C data. Each library is sequenced about 20 million read pairs, and libraries are combined to call loops. The number of loops is called from 1 library to 8 libraries-combined promoter capture Micro-C at 5 kb resolution. (C) Chromatin interaction significance from promoter capture Micro-C (Chicago score, -log *q*-value) of promoter loop categories are shown. Loops are called using promoter capture Micro-C data (182 million read pairs) at 5 kb resolution. (D) Average H3K4me3 ChIP-seq signals around promoters that are in loop vs not in loop. (E) Chromatin accessibility levels (%) of promoters that are in loops vs not in loop are shown. **Figure S7.** Example virtual 4C profiles of Micro-C and promoter capture Micro-C. Using virtual 4C, chromatin interaction signals of Micro-C 3 billion data (top) and promoter capture Micro-C 182 million data (middle) were plotted near three example loop regions at 10 kb resolution (chr1:12120000–12275000, chr1:182600000–182785000, chr7:100900000–100910000). The loop anchoring point is shown in the middle as a red line, and the other interacting region of the loop is highlighted in blue. Refseq genes are shown at the bottom. **Figure S8.** Nucleosome phasing and ChIP-seq signal analysis for regulatory elements and NDRs in loop vs not in loop using Micro-C 3 billion data. Average (A) Micro-C signals, (B) chromatin accessibility scores (%), (C) DNA methylation levels (%) around randomly shuffled (10 times) active promoters, enhancers, insulators, and NDRs without features in loop (black) vs not in loop (orange) are shown. (D) Average ChIP-seq signals around active promoters (H3K4me3 ChIP-seq), enhancers (H3K27ac ChIP-seq), and insulators (CTCF ChIP-seq) in loop (black) vs not in loop (orange) are shown. (D) Average ChIP-seq signals around randomly shuffled (10 times) active promoters (H3K4me3 ChIP-seq), enhancers (H3K27ac ChIP-seq), and insulators (CTCF ChIP-seq) in loop (black) vs not in loop (orange) are shown. (F) Gene expression levels (FPKM) for active promoters in loop vs not in loop are shown. A mean value is shown in red, and median value is shown in blue.

**Additional file 3: Table S2.** Topologically Associating Domains (TADs) identified from this study. (A) TAD statistics of Hi-C, Micro-C, and promoter capture Micro-C data. TAD genomic coordinates of (B) Hi-C 1 billion data, (C) Micro-C 1 billion data, (D) Micro-C 2 billion data, (E) Micro-C 3 billion data, and (F) promoter capture Micro-C data.

**Additional file 4: Table S3.** Chromatin loops identified from this study. (A) summary table of loops called using Mustache (B) summary table of loops called using SIP (C) summary table of loops called using HiCCUPS (D) summary table of loops called using Chicago (E) summary table of neoloops and structural variants called using NeoLoopFinder (F) genomic coordinates of loops found from Hi-C 1 billion data (G) genomic coordinates of loops found from Micro-C 1 billion data (H) genomic coordinates of loops found from Micro-C 2 billion data (I) genomic coordinates of loops found from Micro-C 3 billion data (J) genomic coordinates of loops found from promoter capture Micro-C data (K) genomic coordinates of structural variants and neoloops found from Hi-C 1 billion data (L) genomic coordinates of structural variants and neoloops found from Micro-C 1 billion data (M) genomic coordinates of structural variants and neoloops found from Micro-C 2 billion data (N) genomic coordinates of structural variants and neoloops found from Micro-C 3 billion data.

**Additional file 5: Table S4.** Regulatory elements and nucleosome-depleted regions (NDRs) identified from this study. (A) Number of regulatory elements and NDRs identified from this study (B) genomic coordinates of active promoters, (C) enhancers, (D) insulators, (E) NDRs without features, (F) repressed regions, and (G) heterochromatin regions.

**Additional file 6: Table S5.** Number of loop categories identified by Hi-C, Micro-C, and promoter capture Micro-C data.

## Data Availability

The accession number for the Hi-C, Micro-C, promoter capture Micro-C, NOMe-seq, and RNA-seq data reported in this paper is GEO: GSE205000. The in-house scripts of bioinformatics analysis are available in https://github.com/rhielab/3Dgenome.

## Abbreviations

3C: Chromatin conformation capture
3D: Three-dimensional
ATAC-seq: Assay of transposase accessible chromatin sequencing
ChIP-seq: Chromatin immunoprecipitation sequencing
MNase: Micrococcal nuclease
NDRs: Nucleosome depleted regions
NOMe-seq: Nucleosome occupancy and methylome sequencing
TADs: Topologically associating domains
TSSs: Transcription start sites
