# Correction: Characterizing chromatin interactions of regulatory elements and nucleosome positions, using Hi‑C, Micro‑C, and promoter capture Micro‑C

**DOI:** 10.1186/s13072-023-00498-3

**Published:** 2023-06-07

**Authors:** Beoung Hun Lee, Zexun Wu, Suhn K. Rhie

**Affiliations:** Department of Biochemistry and Molecular Medicine and the Norris, Comprehensive Cancer Center, Keck School of Medicine, University, of Southern California, Los Angeles, CA 90089 USA

**Correction: Epigenetics & Chromatin (2022) 15:41** **https://doi.org/10.1186/s13072-022-00473-4**

The original version of this article [1], unfortunately contained a mistake. The presentation of Fig. 5D has been published incorrectly without Y-axis label. The correct Fig. 5 is provided.

**Fig. 5:**
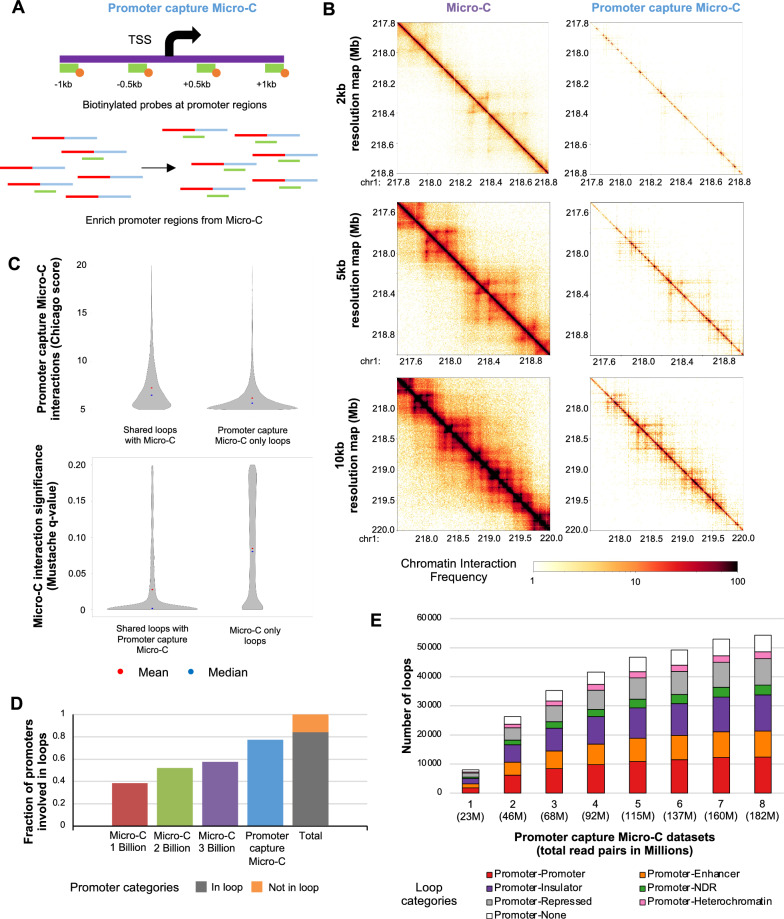
Promoter capture Micro-C data analysis. **A** An overview of promoter capture Micro-C experimental procedure, including the promoter probe design scheme. Probes (green bar) with biotins (orange circle) are designed surrounding TSSs, and Micro-C reads are pulled down using the probes for promoter capture Micro-C. **B** Chromatin interaction heatmaps of Micro-C and promoter capture Micro-C data near chr1q41 region at 2 kb (top), 5 kb (middle), and 10 kb (bottom) resolutions. **C** Significance of chromatin interaction (Chicago score (− log *p*-value), Mustache (*q*-value)) for loops found in both promoter capture Micro-C and Micro-C (shared) and only one data is plotted. A mean value in shown in red. A median value is shown in blue. **D** Fractions of active promoters that intersect with the loop anchors from Micro-C 1 billion, 2 billion, 3 billion data or promoter capture Micro-C data are shown (left). A fraction of active promoters that intersect with loop anchors from any datasets is shown in grey (in loop) while the one not in loop is shown in orange (not in loop) (right). **E** Numbers of promoter-involved loops and loop categories (red: active promoter–active promoter, orange: active promoter–active enhancer, purple: active promoter–active insulator, green: active promoter–NDRs, grey: active promoter–repressed region, pink: active promoter–heterochromatin region, and white: active promoter–none) identified from promoter capture Micro-C data are shown. Loops are called at 5 kb resolution

The original article has been corrected.
