# Supplementary material for: Characterizing chromatin interactions of regulatory elements and nucleosome positions, using Hi-C, Micro-C, and promoter capture Micro-C
Source: Epigenetics Chromatin. 2022 Dec 21;15:41. doi: 10.1186/s13072-022-00473-4 (PMC9768916; doi:10.1186/s13072-022-00473-4)
Supplement: Supplementary file 2 — Additional file 2: Figure S1. Example chromatin interaction heatmaps of Hi-C 1 billion data and Micro-C 1 billion data at high resolutions. (top) 1 kb resolution, (middle) 2 kb resolution, and (bottom) 4 kb resolution. Figure S2. Chromatin loops identified using Hi-C and Micro-C data. Numbers of loops identified by (A) SIP and (B) HiCCUPS loop calling programs at different resolutions (50 kb, 25 kb, 10 kb, 5 kb, 2 kb, and 1 kb resolutions) from Hi-C 1 billion, Micro-C 1 billion, Micro-C 2 billion, and Micro-C 3 billion data. (C) Fractions of loops shared among Mustache, SIP, and HiCCUPS loop calling programs. (D) Distribution of the number of loops at different lengths (distances) for Hi-C 1 billion, Micro-C 1 billion, 2 billion and 3 billion data (loops are called at 10 kb resolution). (E) Distribution of the number of loops at different lengths (distances) for Hi-C 1 billion, Micro-C 1 billion, 2 billion and 3 billion data (loops are called at 5 kb resolution). (F) The numbers of loops shared (between any datasets) among Hi-C 1 billion, Micro-C 1 billion, 2 billion, and 3 billion data are plotted. Shared loops were identified with following priority comparisons; Micro-C 3 billion, Micro-C 2 billion, Micro-C 1 billion, Hi-C 1 billion data. (G) An upset plot of Micro-C 3 billion loops showing the number of loops shared with loops identified from different datasets (Hi-C 1 billion, Micro-C 1 billion, Micro-C 2 billion data). (H) Distribution of the number of unique loops at different lengths (distances) for Hi-C 1 billion, Micro-C 1 billion, 2 billion, and 3 billion data (loops are called at 10 kb resolution). (I) Distribution of the number of unique loops at different lengths (distances) for Hi-C 1 billion, Micro-C 1 billion, 2 billion, and 3 billion data (loops are called at 5 kb resolution). (J) Distribution of the number of shared loops at different lengths (distances) for Hi-C 1 billion, Micro-C 1 billion, 2 billion, and 3 billion data (loops are called at 10 kb [file 13072_2022_473_MOESM2_ESM.docx]

**Supplementary Figure 1. Example chromatin interaction heatmaps of Hi-C 1 billion data and Micro-C 1 billion data at high resolutions.** (top) 1kb resolution, (middle) 2kb resolution, and (bottom) 4kb resolution.

**Supplementary Figure 2. Chromatin loops identified using Hi-C and Micro-C data**. Numbers of loops identified by (A) SIP and (B) HiCCUPS loop calling programs at different resolutions (50kb, 25kb, 10kb, 5kb, 2kb, and 1kb resolutions) from Hi-C 1 billion, Micro-C 1 billion, Micro-C 2 billion, and Micro-C 3 billion data. (C) Fractions of loops shared among Mustache, SIP, and HiCCUPS loop calling programs. (D) Distribution of the number of loops at different lengths (distances) for Hi-C 1 billion, Micro-C 1 billion, 2 billion and 3 billion data (loops are called at 10kb resolution). (E) Distribution of the number of loops at different lengths (distances) for Hi-C 1 billion, Micro-C 1 billion, 2 billion and 3 billion data (loops are called at 5kb resolution). (F) The numbers of loops shared (between any datasets) among Hi-C 1 billion, Micro-C 1 billion, 2 billion, and 3 billion data are plotted. Shared loops were identified with following priority comparisons; Micro-C 3 billion, Micro-C 2 billion, Micro-C 1 billion, Hi-C 1 billion data. (G) An upset plot of Micro-C 3 billion loops showing the number of loops shared with loops identified from different datasets (Hi-C 1 billion, Micro-C 1 billion, Micro-C 2 billion data). (H) Distribution of the number of unique loops at different lengths (distances) for Hi-C 1 billion, Micro-C 1 billion, 2 billion, and 3 billion data (loops are called at 10kb resolution). (I) Distribution of the number of unique loops at different lengths (distances) for Hi-C 1 billion, Micro-C 1 billion, 2 billion, and 3 billion data (loops are called at 5kb resolution). (J) Distribution of the number of shared loops at different lengths (distances) for Hi-C 1 billion, Micro-C 1 billion, 2 billion, and 3 billion data (loops are called at 10kb resolution). (K) Distribution of the number of shared loops at different lengths (distances) for Hi-C 1 billion, Micro-C 1 billion, 2 billion, and 3 billion data (loops are called at 5kb resolution).

**Supplementary Figure 3. Example chromatin interaction heatmaps of Micro-C 3 billion data near loops called at 1kb resolution.** (left) shown is a heatmap at chr7:87,200,000-87,700,000 (middle) shown is a heatmap at chrX:9,800,000-10,300,000 (right) shown is a heatmap at chr9:36,000,000-36,500,000

**Supplementary Figure 4. The number of loops belong to different loop categories.** (A) Hi-C 1 billion data at 5kb resolution, (B) Micro-C 1 billion data at 5kb resolution, (C) Micro-C 2 billion data at 5kb resolution, and (D) Micro-C 3 billion data at 1kb resolution. They are in rank order with the most frequent category at the top and the 28th most frequent category at the bottom.

**Supplementary Figure 5. Detailed analysis of chromatin loop categories identified from Hi-C and Micro-C data.** (A) Statistical significance of chromatin interactions (q-value calculated using Mustache) of Hi-C 1 billion data (left), Micro-C 1 billion data (middle), and Micro-C 2 billion data (right) for top 5 most frequent loop categories. (B) Gene expression levels of active promoters belong to different loop categories. Loops are called using Micro-C 3 billion data at 5kb resolution. Statistical significance (p-value) of gene expression level differences among groups are measured by performing Student’s t-test. Distribution of ChIP-seq signals for (C) active promoter (H3K4me3) loop categories, (D) active enhancer (H3K27ac) loop categories, (E) active insulator (CTCF) loop categories, (F) repressed region (H3K27me3) loop categories, and (G) heterochromatin region (H3K9me3) loop categories. (H) Distribution of chromatin accessibility signals for NDR loop categories. A mean value is shown in red, and median value is shown in blue.

**Supplementary Figure 6. Detailed analysis of promoter capture Micro-C data. (**A) Genome browser screenshots of ChIP-seq, NOMe-seq, promoter capture Micro-C, and Micro-C data near the *STEAP2* gene. (B) Number of loops identified from promoter capture Micro-C data. Each library is sequenced about 20 million read pairs, and libraries are combined to call loops. The number of loops is called from 1 library to 8 libraries-combined promoter capture Micro-C at 5kb resolution. (C) Chromatin interaction significance from promoter capture Micro-C (Chicago score, -log q-value) of promoter loop categories are shown. Loops are called using promoter capture Micro-C data (182 million read pairs) at 5kb resolution. (D) Average H3K4me3 ChIP-seq signals around promoters that are in loop vs not in loop. (E) Chromatin accessibility levels (%) of promoters that are in loops vs not in loop are shown.

**Supplementary Figure 7. Example virtual 4C profiles of Micro-C and promoter capture Micro-C.** Using virtual 4C, chromatin interaction signals of Micro-C 3 billion data (top) and promoter capture Micro-C 182 million data (middle) were plotted near three example loop regions at 10kb resolution (chr1:12120000-12275000, chr1:182600000-182785000, chr7:100900000-100910000). The loop anchoring point is shown in the middle as a red line, and the other interacting region of the loop is highlighted in blue. Refseq genes are shown at the bottom.

**Supplementary Figure 8. Nucleosome phasing and ChIP-seq signal analysis for regulatory elements and NDRs in loop vs not in loop using Micro-C 3 billion data**. Average (A) Micro-C signals, (B) chromatin accessibility scores (%), (C) DNA methylation levels (%) around randomly shuffled (10 times) active promoters, enhancers, insulators, and NDRs without features in loop (black) vs not in loop (orange) are shown. (D) Average ChIP-seq signals around active promoters (H3K4me3 ChIP-seq), enhancers (H3K27ac ChIP-seq), and insulators (CTCF ChIP-seq) in loop (black) vs not in loop (orange) are shown. (D) Average ChIP-seq signals around randomly shuffled (10 times) active promoters (H3K4me3 ChIP-seq), enhancers (H3K27ac ChIP-seq), and insulators (CTCF ChIP-seq) in loop (black) vs not in loop (orange) are shown. (F) Gene expression levels (FPKM) for active promoters in loop vs not in loop are shown. A mean value is shown in red, and median value is shown in blue.
